# Supplementary material for: The Role and the Effect of Magnesium in Mental Disorders: A Systematic Review
Source: Nutrients. 2020 Jun 3;12(6):1661. doi: 10.3390/nu12061661 (PMC7352515; doi:10.3390/nu12061661)
Supplement: Supplementary file 1 [file nutrients-12-01661-s001.pdf]

Supplementary materials

SEARCH SYNTAX:

magnesium AND (psychiatric disorders OR mental diseases OR psychotic disorders OR psychosis OR ultra-high risk psychosis OR schizophrenia OR bipolar disorder OR major depressive disorder OR afffective disorder OR depression OR personality disorder OR anxiety disorder OR obsessive-compulsive disorder OR eating disorders OR ADHD OR autism)

clinical studies:

- *Classical Article*
- *Clinical Study*
- *Clinical Trial*
- *Clinical Trial Protocol*
- *Clinical Trial, Phase I*
- *Clinical Trial, Phase II*
- *Clinical Trial, Phase III*
- *Clinical Trial, Phase IV*
- *Comparative Study*
- *Controlled Clinical Trial*
- *Dataset*
- *Editorial*
- *Evaluation Study*
- *Guideline*
- *Introductory Journal Article*
- *Journal Article*
- *Letter*
- *Multicenter Study*
- *Observational Study*
- *Overall*
- *Practice Guideline*
- *Pragmatic Clinical Trial*
- *Randomized Controlled Trial*
- *Review*
- *Validation Study*

Temporal criterion for strategy: past 10 years.

VARIABLES abstracted for each article included:

- *General information (with sub-columns)*
  - *Title*
  - *First author*
  - *Country of origin*
  - *Date of publication*
  - *Publication status (preprint record, published on peer-reviewed journal)*
- *Study design*
- *Clinical topics covered by the study (with sub-columns)*
  - *psychiatric disorders"/ "mental diseases",*
  - *"psychotic disorders"/ "psychosis"/ "ultra-high risk psychosis"/ "schizophrenia",*
  - *"bipolar disorder"*
  - *"major depressive disorder"/ "affective disorder"/ "depression",*
  - *"personality disorder"*
  - *"anxiety disorders"*
  - *"obsessive compulsive disorders"*
  - *"eating disorders",*
  - *"ADHD"*
  - *"autism"*
